# The Census of 1861 and Lunacy

**Published:** 1860-07-01

**Authors:** 


### ART. VIII.—THE CENSUS OF 1861 AND LUNACY.

WE had hoped that the late Parliamentary Inquiry into the Care and Treatment of Lunatics would have had for one result the institution of an investigation, at the time of the approaching Census, into the amount of lunacy in the kingdom. It might have been thought, that the evidence tendered before the Committee, had made sufficiently plain that it was high time, if we would really deal in a practical manner with the practical question of due provision for our lunatics, we should emerge as quickly as possible from those vague speculations respecting the status of lunacy among us, which bewilder the public and to which, for the want of right data, we are condemned. The increase of *known* lunacy, and particularly of pauperism from lunacy* goes on, seemingly without let or hindrance, at an enormous rate from year to year. We spend money most lavishly in the erection and enlargement of asylums, and yet we barely keep in advance of our most urgent necessities. Nay, were we to do justice to our known lunatics, we have not, even at the present time, with all our efforts, a sufficiency of proper accommodation. The number of pauper lunatics now housed most unfittingly in workhouses, would more than fill all the additional asylum accommodation completed or in progress. Moreover, putting these lunatics entirely out of the question, the accommodation referred to would be entirely occupied in *five* years, supposing that the rate of increase of pauper lunatics in private and public asylums progressed as in the twelve months of 1857.

Again; so hampered are our public asylums with chronic cases of insanity, that with few exceptions our chief asylums have lost completely their character as hospitals for the cure, and have become

* See for the statistics of this question the article *Pauper Lunacy* in our last volume, p. 352.

simply houses for the detention, of lunatics. This result does not arise merely from the gradual accumulation of incurable cases in the asylums, but in a great measure from the rush of chronic and incurable cases to these buildings, as refuges, as soon as they are opened or enlarged.

We cannot tell from the statistics of our asylums in what degree the rapid increase of lunatics in, and the increase of pressure upon, them represent an actual increase of insanity among the population at large, or if they represent such an increase at all. We cannot tell whether the said double-phased increase indicates solely the existence of an unexhausted substratum of chronic lunacy among the people, and yet this is a highly probable supposition. In fact, we are in worse than absolute ignorance of the actual status of lunacy in the kingdom, because we have veneered our knowledge with a mass of statistics, which, while of great value as bearing upon our asylums, have no value as bearing upon the population at large. But, as if gratified by the bulk, and neatness, and formality of these statistics, we persist in making use of them as though they contained all that it was needful to know, and would give an account of all that we ought to know.

How shall we best provide additional accommodation for our lunatics? This is the great question which our Commissioners of Lunacy have to deal with, and which the late Parliamentary Committee ought to settle for us. It is a question that bears at least more heavily on our purses every year, if it will not influence us through a higher medium. Let us glance at the question. First, then, what is the amount of existing lunacy that it is probable we shall have to provide for? Secondly, what is the rate of increase of lunacy among the population at large? The answers to these questions will of course give the chief data for the solution of the problem under consideration. But, alas! the answers are not only not forthcoming, but not even an approximation to them, and we are compelled to have recourse to speculation to supply the data upon which to frame a scheme for meeting a practical need of huge social importance.

Let us look a little further. Supposing that the increase of lunatics in our asylums and poor-houses represents simply a want that we have not been able to overtake; if we get to know the full extent of that want, we have little doubt that the evil it represents would quickly be efficiently compassed.

Supposing that the increase represents an absolute and progressively increasing rate of augmentation of insanity among the population a large; then it is evident that our present means of combating the evil barely even scotch it, and that it is highly necessary we should have some more definite information respecting the fostering causes of lunacy in society.

Under any circumstances, it is certain that the first thing to be done is to institute an inquiry into the amount of lunacy in the kingdom. This, if properly conducted, would give at once a trustworthy basis for subsequent investigations on the progress of insanity among the people, and until this basis be obtained surmise must be substituted for fact. Such an investigation would, moreover, give immediately the data upon which to found a correct opinion on the number of lunatics now at large, for whom provision may probably be required.

The information which we have here set forth to be so urgently needed in this country (and, we may add, in Scotland also), before the vexed question of additional provision for our lunatics can be satisfactorily decided, was obtained for Ireland in 1851, by means of the Census inquiry. Why should not the Census of 1861 be made the means of obtaining for England and Scotland the information specified, the inquiry into the question of lunacy being carried out in the same effective fashion as was done in Ireland? It is to be feared, however, that the Government have determined to let this important question rest, notwithstanding its momentous nature. If this be so, ten years more will probably elapse before the question of the progress of insanity in the kingdom can be put even in the way of being determined; twenty years before the determination can be satisfactorily arrived at. But in the meantime, so rapidly are the questions connected with the status of insanity in the kingdom rising into public prominence, that it would not be surprising if before the termination of the ten years ending in 1871, a special inquiry became necessary, at an enormous expense, to decide, or lay the foundation for deciding, the very question which, if the experience of Ireland may be applied to England, can be readily, and at a comparatively trifling additional expenditure, more efficiently made during the Census inquiry.

